# Clinical Outcome of Rheumatic Mitral Valve Repair and Replacement Surgery in Indonesia; A Comparison with Non-Rheumatic Aetiology

**DOI:** 10.5334/gh.1285

**Published:** 2024-01-11

**Authors:** Amiliana Mardiani Soesanto, Estu Rudiktyo, Amin Tjubandi, Rina Ariani, Fadhila Nafilah Azzahra, Mochammad Faisal Adam, Ario Soeryo Kuncoro

**Affiliations:** 1 Dept. Cardiology and Vascular Medicine, Faculty of Medicine, Universitas Indonesia, National Cardiovascular Center Harapan Kita, Jakarta Barat, DKI Jakarta, Indonesia; 2 Adult Cardiac Surgery Division, Department of Surgery, Faculty of Medicine, Universitas Indonesia/National Cardiovascular Center Harapan Kita, Jakarta Barat, DKI Jakarta, Indonesia; 3 Clinical Research Unit, National Cardiovascular Center Harapan Kita, Indonesia

**Keywords:** Rheumatic Heart Disease, Mitral valve surgery, Survival

## Abstract

**Introduction:**

Mitral valve repair (MVr) has been shown to achieve better outcomes than mitral valve replacement (MVR) in degenerative aetiology. However, that cannot be applied in rheumatic mitral valve disease. Therefore, this study aims to evaluate early and late clinical outcomes and mid-term survival in RHD compared to the non-RHD group and whether mitral valve repair is a better surgical approach in RHD patients.

**Methods:**

Patients who underwent mitral valve surgery with or without coronary artery bypass grafting were included in this study. All patients were divided into the RHD and non-RHD group by the type of mitral surgery performed. Early and late outcomes were evaluated, and mid-term cumulative survival was reported.

**Results:**

A total of 1382 patients post MV surgeries were included. The 30-day mortality was significantly higher in the RHD group compared to the non-RHD group (8.7% vs. 4.4%, p = 0.003). There was no difference in 30-day mortality between repair and replacement in each respective group. During follow-up (12–54 months), all-cause mortality between RHD and non-RHD groups (16.7% vs. 16.2%) was not different. In the RHD group, the survival of MVr was 85.6% (95% CI 82.0%–88.5%), and MVR was 78.3% (95% CI 75.8%–80.6%), p-value log rank 0.26 However, in the non-RHD group, patients who underwent MVr had better survival than MVR, with cumulative survival of 81.7% (95% CI 72.3%–88.2%) vs. 71.1% (95% CI 56.3%–81.7%) p-value log rank 0.007.

**Conclusion:**

Early mortality rate in rheumatic mitral valve surgery was higher than in non-rheumatic valve surgery. Although in rheumatic MV disease MV repair did not show a significant survival advantage over MV replacement, a trend towards more favourable survival in the repair group was observed.

## Introduction

Rheumatic heart disease (RHD) remains an important health problem in developing countries. From a report on the global, regional, and national burden of RHD, the majority of cases come from Asian countries, and Indonesia has become the fourth biggest contributor to global RHD cases [1]. A valvular registry from our country’s biggest tertiary cardiovascular hospital reported that within 3.5 years, 2333 patients with RHD were referred to the hospital, and 94% of the cases involved mitral valve disease [2]. Cost-effective strategy is one consideration among many others to decide which surgical approach will be more suitable, especially in developing countries. Several studies have reported that mitral valve repair is less expensive than replacement in initial and short-term calculations [3,4]. This is because of the use of ring annuloplasty in mitral valve repair (MVr) as opposed to the costly artificial valves, either mechanical or tissue valves, for valve replacement. However, MVr may involve complex techniques and require a longer duration of surgery. It is imperative to carefully assess these two concerns, with the prediction of the clinical outcome, prior to determining the optimal surgical strategy.

Many studies have reported that in degenerative mitral valve disease, mitral valve repair (MVr) achieves better short-term and long-term outcomes than mitral valve replacement (MVR) [5,6]. Further, current guidelines recommend MVr whenever possible [7]. However, in rheumatic mitral valve disease, there is still a controversial issue of whether MVr or MVR is a better surgical approach [8,9,10,11,12,13]. In rheumatic mitral valve disease, the pathology differs from that of degenerative aetiology. There were fibrinoid degeneration, leucocytic infiltrates, Aschoff nodules, calcification, and fibrosis in rheumatic mitral stenosis (MS) histology [14]. In cardiac fibrogenesis, fibroblast proliferation, cellular adhesion, and ECM buildup are stimulated by pro-fibrotic stimuli and activators. The heart valves become calcified and stiffened as a result of a chain reaction, including inflammation and valve fibrosis [15] with commissural and sub-valvar fusion. Most commonly, the mechanism of rheumatic mitral regurgitation (MR) is caused by an elongated anterior leaflet chordae causing prolapse, combined with a retracted posterior leaflet and a dilated mitral annulus [16,17]. These complex pathological changes make repairing valves more challenging [13].

We aim to evaluate the early and late clinical outcome and mid-term survival of mitral valve (MV) surgery in RHD compared to non-RHD in our institution and whether MVr is a better surgical approach for our RHD patients.

## Methods

### Study population

Taking the data from the valvular surgery registry of our hospital, we included all patients with MV disease, age ≥ 18 years, who had undergone MV surgery, with or without coronary artery bypass grafting (CABG) from January 2018 to December 2021. Our institution is the tertiary cardiac center in Indonesia, performing the most valve surgery cases nationwide. Patients with functional MR, concomitant congenital or Bentall surgery, or incomplete data were excluded from the study. All patients were divided based on the aetiology of MV disease into the RHD group and the non-RHD group. The aetiology of valvular heart disease was assessed by echocardiography and confirmed by the surgical finding. The histopathology examination was not performed. Further, each group was divided based on the type of MV surgery into mitral repair and replacement. The decision to repair or replace was decided from the valve surgical conference attended by the heart valve team of our hospital, based on the guidelines of management of valvular heart disease [7,18], and the surgeon’s discretion. Less calcification and less fibrosis of the leaflets and commissure, good anterior leaflet mobility, minimal thickening of the leaflets, and subvalvular apparatus will encourage the surgeons to consider MV repair over replacement in RHD.

The MV surgeries were done mostly by our six senior cardiovascular surgeons, with 10–15 years of experience. On average, each surgeon performed approximately 30–60 MVr/year. Surgeons with more experience and skill in MV repair may confidently perform more repair procedures in rheumatic MV disease, while others prefer replacement in more complicated morphology. The surgical techniques used in repairing the rheumatic MV include commissurotomy, splitting chordae, peeling leaflets, fenestrated chordae, augmentation leaflets with pericardium-treated glutaraldehyde, decalcification/removing leaflets calcium, and restricted chordae resection. While repairing the degenerative MV, the techniques used were triangular resection, quadrangular resection, sliding plasty, folding plasty, chordae transfer, shortening chordae, artificial chordae, commissure-plasty, and edge-to-edge repair. During the surgery, transoesophageal echocardiography (TEE) was done by a cardiac anaesthesiologist to evaluate the result of MV repair/replacement. If TEE showed an unaccepted repair result due to significant residual regurgitation or stenosis, the strategy was converted to replace the valve.

### Outcomes

The study endpoints were early and late outcomes after MV surgery. Early outcomes included mortality and reoperation procedures at any cause within 30 days post-surgery. Late outcomes were any occurrence of all-cause mortality and mitral valve reoperation, including late survival until the end of the follow-up period on 30 June 2022, whichever came first. Mortality was detected by phone calls, a death record, or withdrawal from the National Health Coverage program. In addition, information regarding reoperation was retrieved from our medical record. Our institution’s Research Board Committee granted ethical clearance for this study (no: LB.02.01/VII/026/KEP026/2023).

### Statistics

Baseline characteristics of the patients, including descriptive data of demographics, preoperative comorbidity, concomitant cardiac intervention, and echo parameters of cardiac condition, were compared between MVr versus MVR of each etiologic group, RHD versus non-RHD. Continuous variables were presented as mean ± standard deviation (SD) for normal data distribution or median (minimum-maximum) for abnormal ones. Categorical variables were presented as frequencies and percentages. We compared every two groups using the t-test or the Mann-Whitney U test for continuous normally or non-normally distributed data, respectively, and the *Chi*-square or Fisher’s exact test for categorical data.

Factors associated with 30-day mortality were evaluated by calculating the odds ratios (OR) using *Chi*-square or Fisher’s exact test for bivariate analysis. Any variables with a p-value less than 0.25 were further continued to multivariate analysis, using multiple logistic regression to evaluate any independent correlation with the outcome. A 95% confidence interval applied to estimate the precision of the odds. For evaluating predictors that possibly have any correlation with late mortality, Cox’s proportional hazard model was performed to calculate the hazard ratio (HR). Finally, we assess the cumulative survival of each surgical approach in RHD and non-RHD groups using the Kaplan-Meier curve and Mantel-Cox (log-rank) test to compare differences between groups.

For all statistical calculations, a *p-*value < 0.05 was considered statistically significant. The IBM SPSS Statistics for Windows, version 20 (IBM Corp., Armonk, N.Y., USA) was used for data analysis.

## Result

### Baseline characteristics

**Figure 1:**
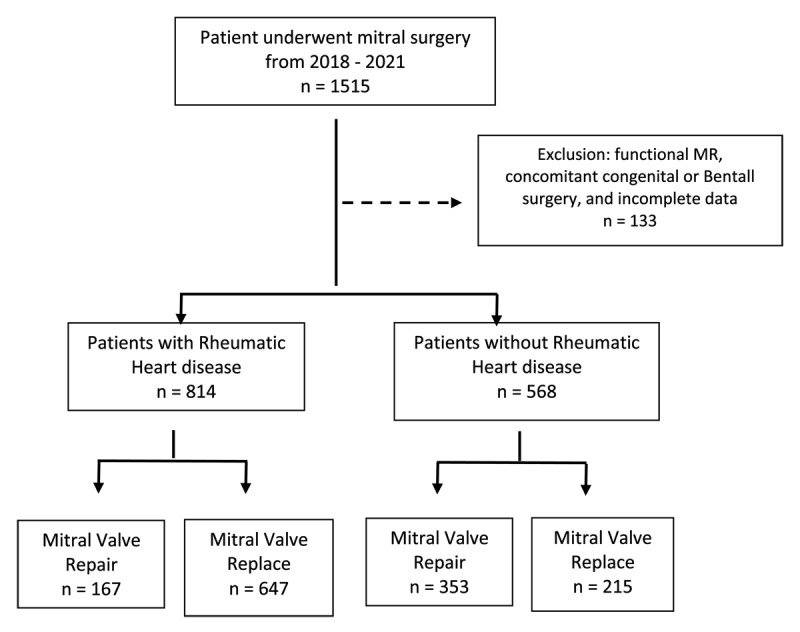
Flow chart of patients’ recruitment based on the aetiology of mitral valve disease and the surgical approach.

A total of 1515 MV surgeries were performed from 2018–2021. After excluding patients with functional MR, concomitant congenital, Bentall surgery, or incomplete data, we included 1382 patients in the study. Patients with rheumatic aetiology were **814 (58.5%) patients**, and MVr was performed in **167 (20.6 %) patients** of the cases. In non-rheumatic aetiology, MVr was done in **353 (61.6%) patients** (Figure 1). In both groups, mechanical prosthetic valves were mostly used for MVR.

The baseline characteristics of rheumatic MV patients differed from those of non-rheumatic patients (Table 1). Younger age, female gender, atrial fibrillation (AF), multiple procedures, longer cardiopulmonary bypass (CPB) and aortic cross-clamp (AoX) time, higher pulmonary artery pressure, lower left ventricular ejection fraction (LVEF), and right ventricular (RV) contractility were found more commonly in rheumatic MV disease. The average Euroscore II was significantly higher in the RHD group **(2.0; SD 0.5–19.5)**, compared to the non-RHD group **(1.6; SD 0.5–21.3)**, with p < 0.001. Within the rheumatic group, AF, tricuspid, and mixed valve surgery were seen more often in patients with MVR. Patients with isolated rheumatic MR and higher tricuspid annular plane systolic excursion (TAPSE) had more MVr. The detailed data with the respective p-values was included in Supplement Table 1.

**Table 1:** Basic characteristics of MV surgery patients based on aetiology and surgical approach.


VARIABLES	RHEUMATIC HEART DISEASE			NON-RHEUMATIC HEART DISEASE		
	
	TOTAL n = 814	MITRAL REPAIR n = 167	MITRAL REPLACE n = 647	TOTAL n = 568	MITRAL REPAIR n = 353	MITRAL REPLACE n = 215

**Demography**						

Age	43 (18–70)	41 (18–69)	43 (18–70)	54 (18–78)*	54 (18–78)	53 (18–73)

Female	515 (63.3%)	112 (67.1%)	403 (62.3%)	182 (32%)*	121 (34.3%)	61 (28.4%)

Body Mass Index	21.9 (13.6–43.4)	22.1 (13.6–39.7)	21.8 (14.0–43.4)	23.1 (12.9–39.1)*	23.5 (12.9–39.1)	22.2(14.5–39.1)‡

**Preoperative Comorbidities**						

Atrial Fibrillation	587 (72.1%)	106 (63.5%)	481 (74.3%)†	180 (31.7%)*	106 (30%)	74 (34.4 %)

DM with Insulin, n (%)	6 (0.7%)	1 (0.6%)	5 (0.8%)	6 (1.1%)	4 (1.1%)	2 (0.9%)

COPD, n (%)	5 (0.6%)	0 (0%)	5 (0.8%)	2 (0.4%)	0 (0%)	2 (0.9%)

Hypertension	81 (10%)	14 (8.4%)	67 (10.4%)	179 (31.5%)*	117 (33.1%)	62 (28.8%)

CKD (CCL< 50 ml/m^2^)	119 (14.6%)	26 (15.6%)	93 (14.4%)	146 (25.7%)*	80 (22.7%)	66 (30.7%)‡

NYHA fc III-IV	402 (49.4%)	76 (45.5%)	326 (50.4%)	254 (44.7%)	151 (42.8%)	103 (47.9%)

Infective Endocarditis	22 (2.7%)	5 (3%)	17 (2.6%)	44 (7.7%)*	10 (2.8%)	34 (15.8%)‡

**Concomitant Cardiac Surgery/Intervention**						

CABG	27 (3.3%)	6 (3.6%)	21 (3.2%)	93 (16.4%)*	48 (13.6%)	45 (20.9%)

Aortic Valve Surgery	238 (29.2%)	46 (27.5%)	192 (29.7%)	40 (7%)*	18 (5.1%)	22 (10.2%)‡

Tricuspid Valve Surgery	472 (58%)	82 (49.1%)	390 (60.3%)†	141 (24.8%)*	60 (17%)	81 (37.7%)‡

≥3 surgical procedures	134 (16.5%)	25 (15%)	109 (16.8%)	16 (2.8%)*	4 (1.1%)	12 (5.6%)‡

History of PTMC	13 (1.6%)	3 (1.8%)	10 (1.5%)	0 (0%)*	0 (0%)	0 (0%)

CPB time	106 (41–390)	109 (44–251)	105.5 (41–390)	100 (10–515)*	95 (10–261)	110 (45–515)‡

Aox time	80 (5–319)	78 (5–225)	80.5 (27–319)	76 (12–467)*	72.50 (12–235)	82 (25–467)‡

**Cardiac condition (echo parameters)**						

LVEF (%)	60 (20–82)	60 (22–80)	60 (20–82)	66 (25–89)*	67 (27–89)	65 (25–82)‡

TAPSE (mm)	18 (6–37)	19 (8–36)	18 (6.0–37.0)†	23 (5.3–40)*	24.0 (6.2–39.4)	22 (5.3–40)

TVG	41 (0–156)	37 (0–100)	42 (0–156)	31 (0–111)*	30 (0–108)	33 (0–111)‡

Mitral stenosis	342 (42%)	69 (41.3%)	273 (42.2%)	0 (0%)*	0 (0%)	0 (0%)

Mitral regurgitation	166 (20.4%)	52 (31.1%)	114 (17.6%)†	568 (100%)*	353 (100%)	215 (100%)

Mixed mitral valve disease	306 (37.6%)	46 (27.5%)	260 (40.2%)†	0 (0%)*	0 (0%)	0 (0%)

**Risk Assessment for Mortality**						

Euroscore II	2.0 (0.5–19.5)	1.9 (0.5–19.5)	2.1 (0.5–16.2) †	1.6 (0.5–21.3)*	1.4 (0.5–14.0)	2.2 (0.5–21.3)‡


DM: diabetes mellitus, COPD: Chronic Obstructive Pulmonary Disease, CKD: chronic kidney disease, NYHA fc: New York Heart Association functional class, CABG: Coronary Artery Bypass Grafting, PTMC: percutaneous transvenous mitral commissurotomy, CPB: cardiopulmonary bypass, AoX: aortic cross-clamp, LVEF: left ventricular ejection fraction, TAPSE: tricuspid annular plane systolic excursion, TVG: tricuspid valve gradient. *Comparing RHD and non-RHD, p < 0.05. *†Comparing repair and replacement in RHD, p < 0.05*. *‡Comparing repair and replacement in non-RHD repair, p < 0.05*.

**Figure 2:**
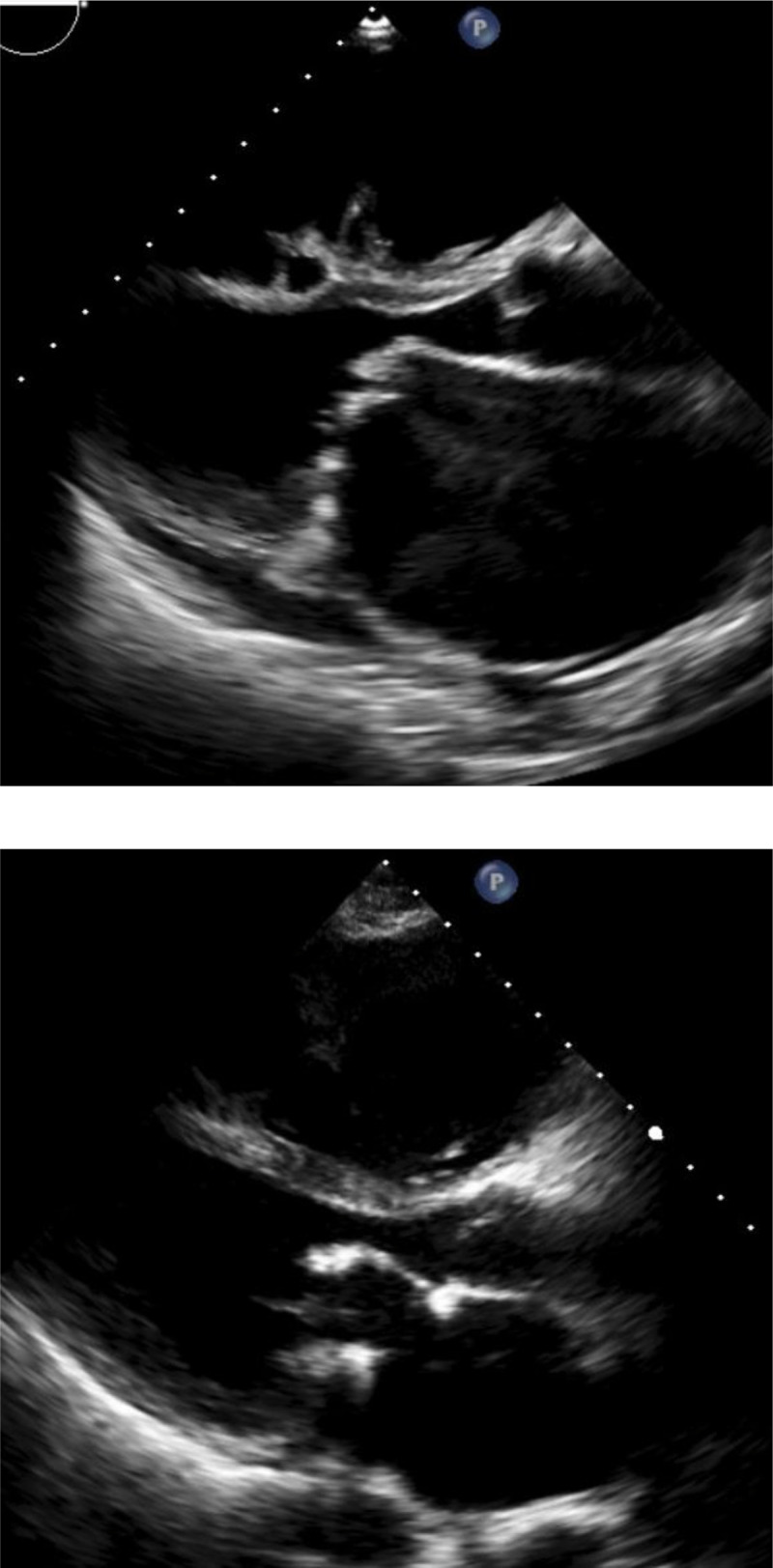
The echocardiographic pictures of rheumatic mitral valve disease pre (above picture) and post repaired (below picture).

**Figure 3:**
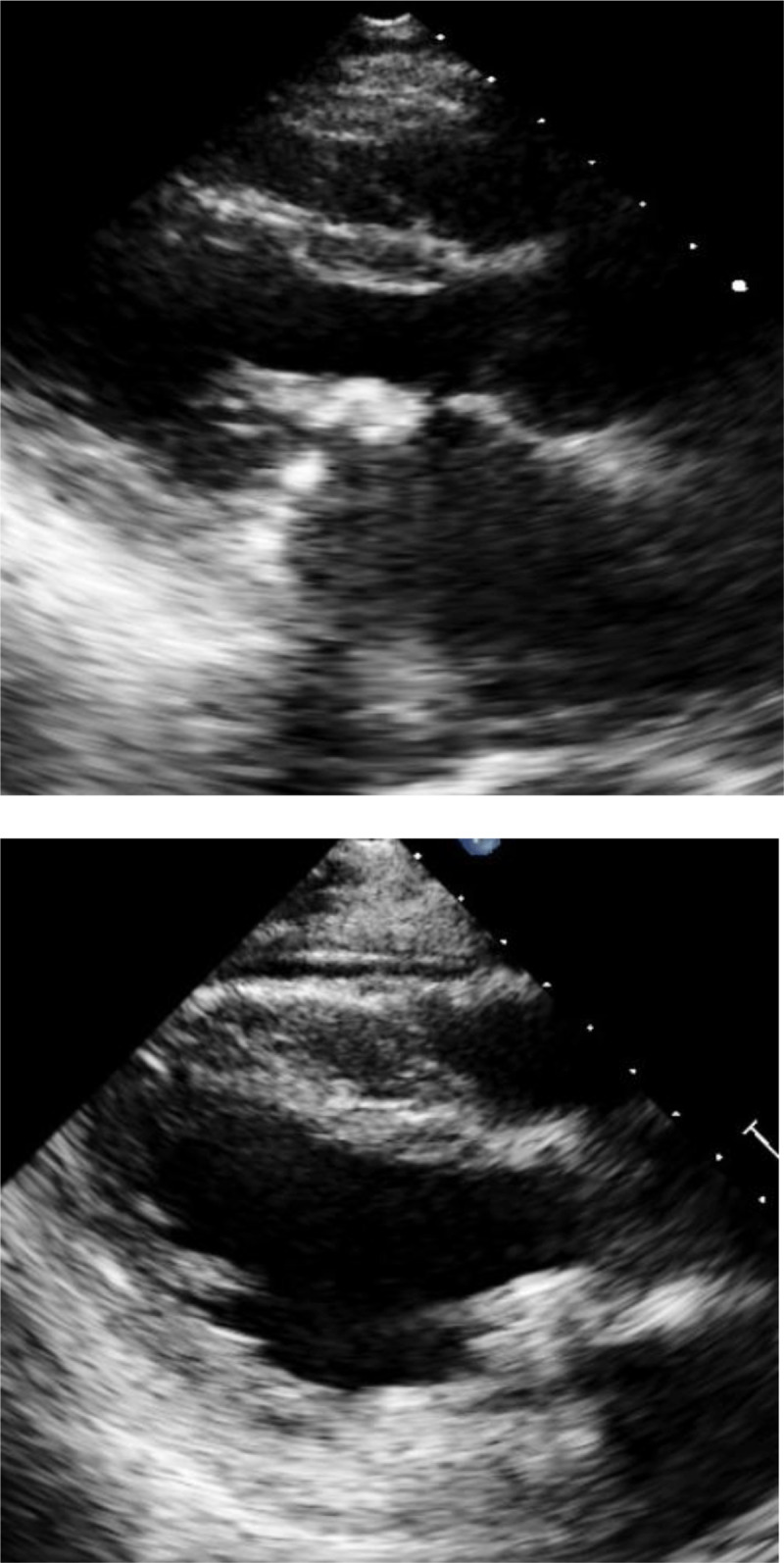
The echocardiographic pictures of rheumatic mitral valve disease pre (above picture) and post replacement with mechanical prosthetic valve (below picture).

The most common type of rheumatic MV disease was MS in **342 (42%) patients**, followed by mixed mitral valve disease in **306 (37.6%) patients**. Rheumatic MV morphology showed thickened and fibrotic leaflet, chordae, and commissure fusion, with some calcification. Figure 2 shows the echocardiographic pictures of rheumatic MV disease pre- and post-repaired. Figure 3. shows the echocardiographic pictures of rheumatic MV disease pre- and post-replacement with a mechanical prosthetic valve. Non-rheumatic MV aetiology was degenerative myxomatous in **553 (97.3%) patients**, presenting with redundant leaflets, prolapse, flail, with or without chordae rupture. Other aetiologies were degenerative calcification and others in **15 (2.6%) patients**, including infective endocarditis and a patient with a history of MVr.

### Early and late outcome

As an early outcome, the 30-day mortality was significantly higher in the rheumatic group compared to the non-rheumatic group, with **71 (8.7%) patients** and **25 (4.4%) patients** (p = 0.003), respectively. The three most common causes of death in the RHD group were cardiac cause in **53 (6.5%) patients**, neurologic cause **in 5 (0.6%) patients**, and infection/sepsis in **5 (0.6%) patients**. While in the non-RHD group, the three most common causes of death were cardiac causes in **13 (2.3%) patients**, lung causes in **5 (0.9%) patients**, and infection/ sepsis in **2 (0.4%) patients**. There was no difference in 30-day mortality between MVr and MVR approaches within each etiologic group. Reoperation within 30 days was performed in **15 (1.8%) cases** in the rheumatic group and **9 (1.6%) cases** in the non-rheumatic group without a statistically significant difference, as shown in Table 2. The causes of reoperation within 30 days in both groups were bleeding in **15 (62.5%) cases** and tamponade **in 5 (20.8%) cases**. The detailed data with the respective p-values was included in Supplement Table 2.

**Table 2:** Outcome following mitral valve surgery based on aetiology and surgical approach.


VARIABLES	RHEUMATIC HEART DISEASE			NON-RHEUMATIC HEART DISEASE		
	
	TOTAL n = 814	MITRAL REPAIR n = 167	MITRAL REPLACE n = 647	TOTAL n = 568	MITRAL REPAIR n = 353	MITRAL REPLACE n = 215

**Early outcome**						

30-day mortality	71 (8.7%)	14 (8.4%)	57 (8.8%)	25 (4.4%)*	12 (3.4%)	13 (6.0%)

30-day reoperation	15 (1.8%)	4 (2.4%)	11 (1.7%)	9 (1.6%)	6 (1.7%)	3 (1.4%)

**Late outcome**						

Late all-cause mortality	136 (16.7%)	22 (13.2%)	114 (17.6%)	92 (16.2%)	46 (13%)	46 (21.4%)‡

Late MV reoperation	3 (0.4%)	0 (0%)	3 (0.5%)	5 (0.9%)	4 (1.1%)	1 (0.5%)


*MV: mitral valve*. * Comparing RHD and non-RHD, p < 0.05. *† Comparing repair and replacement in RHD, p < 0.05*. *‡ Comparing repair and replacement in non-RHD repair, p < 0.05*.

We followed the patients with a median follow-up time of 34 months (12–54), and **21 (1.5%) patients** were lost of follow-up. Late outcomes were MV reoperation and all-cause mortality (Table 2). At the end of the follow-up period, mortality occurred in **136 (16.7%) patients** in the rheumatic group and **92 (16.2%) patients** in the non-rheumatic group without any significant difference. In the rheumatic group, the late mortality of MVr and MVR occurred in **22 (13.2%) and 114 (17.6%) cases**, respectively, without significant differences. On the contrary, in the non-rheumatic group, late mortality in MVr was significantly lower than MVR, which was **46 (13%) vs. 46 (21.4%) patients**, respectively (p = 0.012). There were just a few MV reoperations during the follow-up, with 8 (0.58%) cases, without a significant difference between the rheumatic and non-rheumatic MV groups. In MVr patients, late reoperation was performed due to severe MR, and in MVR patients, the reasons for late reoperation were prosthetic malfunction (thrombosis) and infective endocarditis.

The factors associated with early and late mortality in the rheumatic group were evaluated. Bivariate analysis showed that older age than 60 years, AF, New York Heart Association functional class (NYHA fc) III–IV, and Euroscore II over 4 may increase the risk for 30-day mortality in the RHD group. However, from the multivariate analysis, only AF and Euroscore ≥ 4 were the independent predictors for 30-day mortality (Table 3). For the non-RHD group, age, AF, NYHA fc III-IV, and ≥ 3 surgical procedures were the independent predictors for 30-day mortality (Table 4).

**Table 3:** Factors associated with 30-day mortality after rheumatic mitral valve surgery.


VARIABLE	UNIVARIATE			MULTIVARIATE		
	
	OR	95%CI	p-VALUE	OR	95%CI	p-VALUE

Age > 60 Years	2.82	1.25–6.38	0.018*			

Female	0.60	0.37–0.98	0.056	0.55	0.33–0.91	0.020*

BMI > 25.0 kg/m^2^	0.53	0.28–1.04	0.082	0.53	0.27–1.04	0.064

Atrial Fibrillation	2.86	1.40–5.86	0.004*	2.86	1.39–5.89	0.004*

Hypertension	0.99	0.44–2.24	1.00			

NYHA fc III–IV	1.75	1.06–2.89	0.036*			

Infective Endocarditis	1.05	0.24–4.58	1.00			

Concomitant AVR	1.26	0.75–2.12	0.454			

Concomitant TVr	1.37	0.82–2.28	0.276			

Concomitant CABG	2.48	0.91–6.77	0.078			

≥3 surgical procedures	1.41	0.77–2.57	0.346			

History of PTMC	1.93	0.42–8.88	0.316			

LVEF < 30%	1.76	0.21–14.78	0.473			

TAPSE < 17 mm	1.47	0.90–2.39	0.155			

TVG ≥ 50 mmHg	0.91	0.55–1.51	0.811			

Mitral Stenosis	1.47	0.90–2.39	0.154			

Mitral Regurgitation	0.70	0.36–1.36	0.358			

Mixed MV Disease	0.84	0.50–1.40	0.574			

Mitral valve Repair	0.95	0.51–1.75	0.984			

Euroscore II > 4	3.32	1.70–6.49	0.001*	3.86	1.93–7.74	<0.0001*


BMI: body mass index, AVR: Aortic Valve Replacement, TVr: Tricuspid Valve repair, CABG: Coronary Artery Bypass Grafting, PTMC: percutaneous transvenous mitral commissurotomy, LVEF: Left Ventricular Ejection Fraction, TVG: Tricuspid Valve Gradient. Data were analysed using the Chi-Square Test and Logistic Regression for the multivariate analysis. * Statistically significant (p < 0.05).

**Table 4:** Factors associated with 30-day mortality after non-rheumatic mitral valve surgery.


VARIABLE	UNIVARIATE			MULTIVARIATE		
	
	OR	95%CI	p-VALUE	OR	95%CI	p-VALUE

Age > 60 Years	3.36	1.48–7.61	0.005*	3.06	1.24–7.53	0.015*

Female	0.52	0.19–1.40	0.271			

BMI > 25.0 kg/m^2^	0.17	0.04–0.72	0.012*	0.22	0.05–0.95	0.042*

Atrial Fibrillation	4.11	1.78–9.49	0.001*	3.06	1.25–7.48	0.014*

COPD	22.58	1.37–372.04	0.086			

CKD (CCL < 50 ml/m^2^)	2.38	1.05–5.36	0.057			

NYHA fc III-IV	2.28	0.99–5.25	0.075	2.65	1.06–6.65	0.038*

Infective Endocarditis	1.04	0.24–4.55	1.000			

Concomitant AVR	1.87	0.53–6.52	0.409			

Concomitant TVr	2.96	1.32–6.65	0.012*			

Concomitant CABG	2.07	0.84–5.10	0.160			

≥3 surgical procedures	16.83	5.54–51.11	<0.001*	15.57	4.46–54.31	<0.001*

TAPSE < 17 mm	1.31	0.44–3.93	0.548			

TVG ≥ 50 mmHg	1.75	0.74–4.16	0.300			

Mitral valve Repair	0.55	0.25–1.22	0.200			

Euroscore II > 4	3.86	1.36–10.95	0.020*			


BMI: body mass index, AVR: Aortic Valve Replacement, TVr: Tricuspid Valve repair, CABG: Coronary Artery Bypass Grafting, PTMC: percutaneous transvenous mitral commissurotomy, LVEF: Left Ventricular Ejection Fraction, TVG: Tricuspid Valve Gradient. Data were analysed using the Chi-Square Test and Logistic Regression for the multivariate analysis. * Statistically significant (p < 0.05).

Further, we evaluated factors associated with late mortality in the rheumatic group (Table 5). In the RHD group, age over 60 years, AF, NYHA fc III-IV, concomitant aortic valve replacement (AVR), and Euroscore ≥ 4 were independent predictors for late mortality. Mitral valve repair was not protective of late mortality with HR 0.77 (95% CI 0.49–1.22, p = 0.266). While in the non-RHD group, as shown in Table 6, MVr was the protective factor for late mortality. The independent predictors for late mortality were AF, CKD, NYHA fc III-IV, and ≥ 3 surgical procedures.

**Table 5:** Factors associated with late mortality in patients with rheumatic MV surgery.


VARIABLE	UNIVARIATE			MULTIVARIATE		
	
	HR	95%CI	p-VALUE	HR	95%CI	p-VALUE

Age > 60 Years	2.72	1.56–4.74	<0.001*	2.17	1.23–3.81	0.007*

Female	0.57	0.41–0.80	<0.001*	0.58	0.41–0.83	0.002*

BMI > 25.0 kg/m^2^	0.91	0.61–1.35	0.633			

Atrial Fibrillation	3.27	1.91–5.59	<0.001*	3.42	2.0–5.87	<0.001*

DM with Insulin. n (%)	1.99	0.49–8.03	0.335			

Hypertension	0.96	0.54–1.70	0.881			

NYHA fc III-IV	1.78	1.25–2.54	0.001*	1.62	1.13–2.32	0.008*

Infective Endocarditis	0.74	0.24–2.32	0.602			

Concomitant AVR	1.59	1.13–2.25	0.008*	1.60	1.12–2.28	0.010 *

Concomitant TVr	1.03	0.74–1.45	0.850			

Concomitant CABG	1.19	0.49–2.91	0.702			

≥3 surgical procedures	1.56	1.04–2.35	0.031*			

History of PTMC	1.97	0.73–5.32	0.182	2.70	0.99–7.39	0.053

LVEF < 30%	2.17	0.54–8.79	0.276			

TAPSE < 17 mm	1.34	0.96–1.88	0.086			

TVG ≥ 50 mmHg	0.93	0.65–1.32	0.687			

Mitral Stenosis	1.22	0.87–1.70	0.258			

Mitral Regurgitation	0.85	0.55–1.32	0.470			

Mixed mitral Valve Disease	0.91	0.64–1.29	0.586			

Mitral valve Repair	0.77	0.49–1.22	0.266			

Euroscore II > 4.0	2.56	1.59–4.12	<0.001*	2.08	1.26–3.44	0.004*


BMI: body mass index, AVR: Aortic Valve Replacement, TVr: Tricuspid Valve repair, CABG: Coronary Artery Bypass Grafting, PTMC: percutaneous transvenous mitral commissurotomy, LVEF: Left Ventricular Ejection Fraction, TVG: Tricuspid Valve Gradient. Data were analysed by Cox Regression. *Statistically significant (p-value < 0.05).

**Table 6:** Factors associated with late mortality in patients with non-rheumatic MV surgery.


VARIABLE	UNIVARIATE			MULTIVARIATE		
	
	HR	95%CI	p-VALUE	HR	95%CI	p-VALUE

Age > 60 Years	1.86	1.19–2.90	0.006*	1.50	0.95–2.38	0.085

Female	0.55	0.33–0.91	0.020*	0.56	0.33–0.94	0.029*

BMI > 25.0 kg/m^2^	0.68	0.42–1.07	0.097			

Atrial Fibrillation	2.75	1.82–4.14	<0.001*	2.31	1.51–3.52	<0.001*

COPD	3.50	0.49–25.19	0.214			

CKD (CCL < 50 ml/m^2^)	1.92	1.26–2.92	0.002*	1.58	1.01–2.48	0.046*

NYHA fc III-IV	2.12	1.38–3.27	0.001*	2.27	1.47–3.53	<0.0001*

Infective Endocarditis	0.38	0.12–1.18	0.094	0.37	0.11–1.18	0.092

Concomitant AVR	1.86	0.99–3.49	0.054			

Concomitant TVr	1.86	1.20–2.87	0.005*			

Concomitant CABG	1.66	1.03–2.67	0.039*			

≥3 surgical procedures	6.06	3.03–12.13	<0.001*	3.97	1.93–8.18	<0.001*

TAPSE < 17 mm	1.86	1.13–3.09	0.016*			

TVG ≥ 50 mmHg	1.15	0.71–1.88	0.566			

Mitral valve Repair	0.58	0.38–0.87	0.009*	0.65	0.42–0.99	0.047*

Euroscore II > 4	2.76	1.53–4.96	0.001*			


BMI: body mass index, AVR: Aortic Valve Replacement, TVr: Tricuspid Valve repair, CABG: Coronary Artery Bypass Grafting, PTMC: percutaneous transvenous mitral commissurotomy, LVEF: Left Ventricular Ejection Fraction, TVG: Tricuspid Valve Gradient. Data were analysed by Cox Regression. *Statistically significant (p-value < 0.05).

Separately, we evaluated the association between 30-day reoperation and late mortality and found that in both RHD and non-RHD groups, the 30-day reoperation significantly affected late mortality, with HR 5.02 (95% CI 2.63–9.58) p < 0.001 in the RHD group and HR 5.89 (95% CI 2.38–14.59) p < 0.001 in the non-RHD group (Table 3).

### Survival

**Figure 4:**
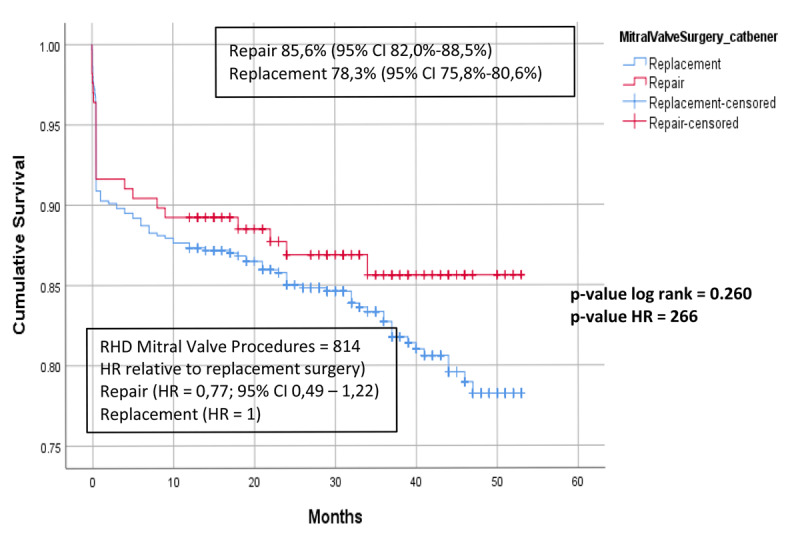
Cumulative survival, comparison between MVr and MVR in the RHD group.

**Figure 5:**
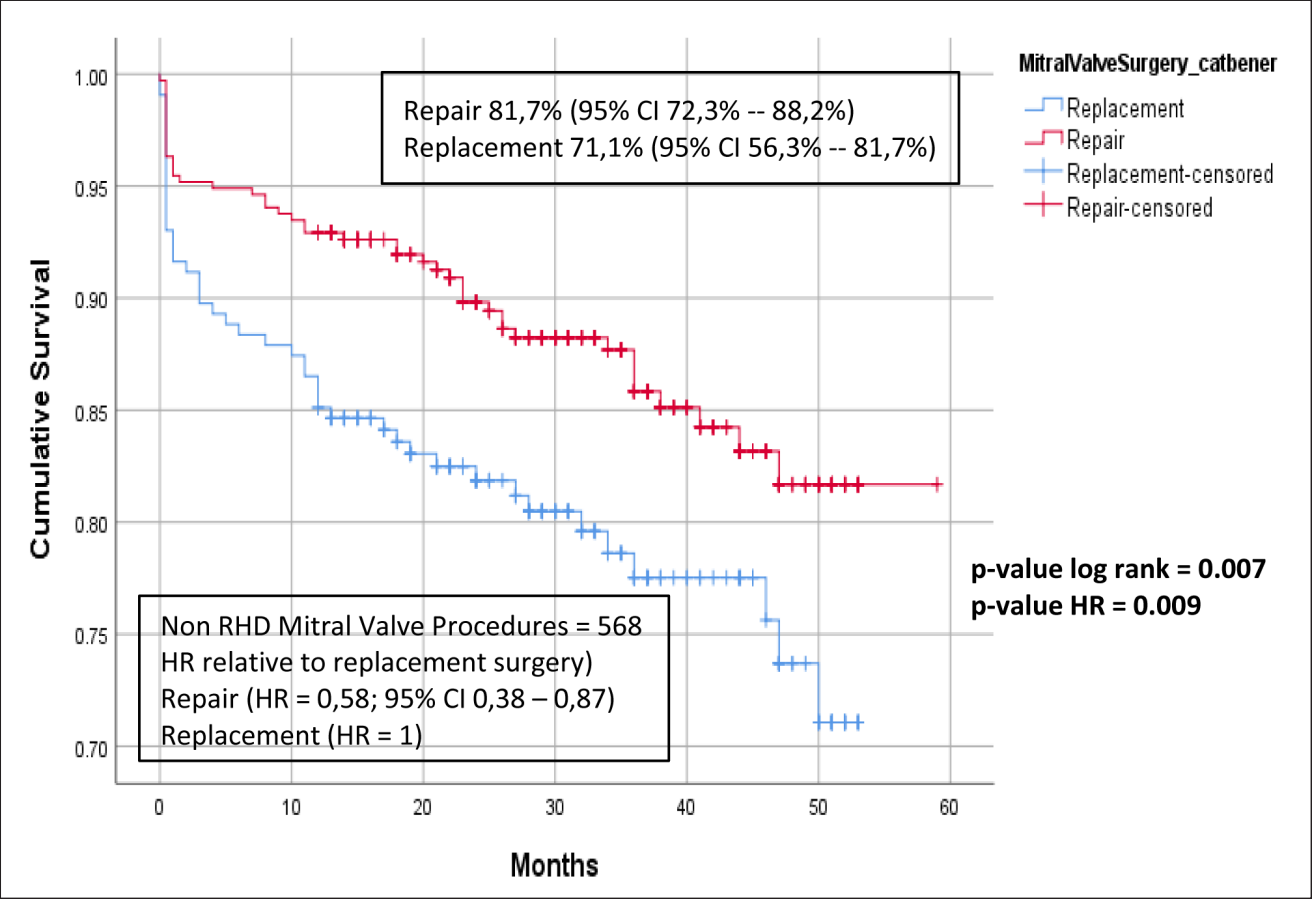
Cumulative survival, comparison between MVr and MVR in the non-RHD group.

In the rheumatic group, there was no statistical difference between the survival of patients who underwent MVr was 85.6% (95% CI 82.0%–88.5%) and those who underwent MVR 78.3% (95% CI 75.8%–80.6%) p-value log rank 0.26 (Figure 4). However, in the non-rheumatic group, patients who underwent MVr had better survival than MVR, with cumulative survival of 81.7% (95% CI 72.3%–88.2%) and 71.1% (95% CI 56.3%–81.7%) respectively, p-value log rank 0.007 (Figure 5).

Comparison between surgical approach in both etiologic groups for early, late mortalities, and cumulative survival was illustrated in the central illustration figure.

## Discussion

This study evaluated the early and late outcomes of MVr and MVR in the RHD patients and compared them with the non-RHD patients. We found that the 30-day mortality in the RHD group was significantly higher than in the non-RHD group, 8.7%, and 4.4%, respectively (p < 0.05). While late mortality was equal in both RHD and non-RHD groups, 16.7% and 16.2%, respectively (p = 0.859). Further, within the RHD group, the 30-day and late mortality were not different between the MVr and MVR groups. A comparable result was reported by Chen et al. with the in-hospital mortality rates of 7.1% and 7.3% in the repair and replacement groups, respectively, without any significant difference [12]. Equal survival was observed between MVr and MVR in our RHD patients, which has also been reported in many studies [9,12,19,20]. In contrast, in the non-RHD patients, we observed that patients with MVr showed a significantly higher survival than MVR, and MVr was independently a protective factor for late mortality. This finding was common as there is strong evidence that in degenerative mitral valve disease, MVr has a better outcome than MVR, establishing recommendations to perform MVr whenever possible [7,21].

Although our RHD patients were more likely to be female, younger, and had lower BMIs than non-RHD patients, they also had more frequent AF, required more surgical procedures, had longer cardiopulmonary bypass and aortic cross-clamp times, had lower LVEF and TAPSE, higher pulmonary artery pressure, and higher Euroscore II scores, which may result in excess mortality when compared to non-RHD patients. We found that in the RHD group, an older age than 60 years, AF, NYHA fc III-IV, and Euroscore > 4 were correlated with an increased risk of 30-day mortality. These conditions have been recognised to carry a higher risk of surgical mortality and were included as variables in Euroscore II and STS scores as cardiac surgical risk scoring system [22,23].

The majority of our mitral surgery cases were rheumatic aetiology. This condition differed from other reports, which were predominated by non-rheumatic aetiology [12,20,24]. The most common lesions in our rheumatic group were isolated MS (41.7%), followed by mixed mitral lesions (33.8%). The valve morphology showed thickened and fibrotic leaflet, chordae, and commissure fusion, with some degree of calcification. Aside from those complex MV morphologies, a significant number of the concomitant aortic valve (29.2%), tricuspid valve (58%), and triple valve (16.5%) surgeries suggested more severe rheumatic processes in our patients, which could affect the surgical strategy. Our surgeons considered repairing the valve with less calcification and less fibrotic of the leaflets and commissure, good anterior leaflet mobility, and minimal thickening of the leaflets and subvalvular apparatus. McCartney, in his commentary article, suggests that valve repair may be the best approach for especially younger patients with pure rheumatic MR and mobile anterior leaflets [25].

We found that AF was significantly more frequent in the RHD group compared to the non-RHD group. Permanent AF was detected in more than 70% of our rheumatic patients and correlated with significant 30-day mortality and late survival. The proportion of AF in our study was higher than those reported in other studies [12,20,26]. Atrial Fibrillation may have influenced the result, as it is known that AF is a complication of severe valvular heart disease [27]. Several studies reported that AF is a predictor for poor outcomes in post-valvular surgery as well as in non-surgical patients [28,29]. Excess mortality and morbidity may be due to a high risk of thromboembolic events, including stroke and bleeding risk as a result of longstanding anticoagulation used. Successful cryoablation surgery (e.g., Cox-Maze procedure) may convert AF to sinus rhythm. Combining the Maze procedure with mitral valve surgery, including in rheumatic aetiology, is safe and effective and improves long-term survival [30,31]. Unfortunately, this procedure is rarely performed in our center because of financial constraints.

We found that the late survival of MVr and MVR was comparable to other studies [11,20]. High volume of annual rheumatic valve surgery in our center might affect the outcome, resulting in a trend of better survival of MVr procedure compared to MVR, which was 85.6% (95% CI 82.0%–88.5%) and 78.3% (95% CI 75.8%–80.6%) respectively, although not statistically significant, p = 0.26. Further, unlike in our non-RHD group, the type of surgical approach, repair, or replacement was not correlated with survival in the RHD group. In our study, reduced survival was linked to specific characteristics, including age over 60 years, AF, CKD, NYHA fc III-IV, concurrent AVR, more than three surgical procedures, and a history of PTMC. Saurav et al. reported that in RHD patients, concomitant aortic valve surgery might reduce the survival advantage of MVr, suggesting that MVR is preferable in the case of double valve surgery [32]. Atrial fibrillation, multiple valve disease, and severe morphology were the typical characteristics of more advanced RHD and showed significant correlations with the late outcome. It could be suggested that the severity of the rheumatic process correlates with the outcome.

Interestingly, although not correlated with the outcome, the LVEF in our RHD group was significantly lower than in our non-RHD group. The same finding was also reported by Rudiktyo et al [33], suggesting that besides the chronic load of the left ventricle, there could be another mechanism that worsens the LV function in patients with rheumatic MR. Other studies reported that the intrinsic myocardial process in RHD may play a role in the mechanism of this impaired LV contractility, as shown in the cardiac MRI and strain echocardiography [34,35]. Reduced LV and RV function, older age, more complex valve lesions, and a high AF proportion suggested that most of our patients presented in their late stages. There were possible reasons: (1) Financial reasons; more patients came from different cities or islands. Although the procedure was covered by national health coverage, they must have had enough financial support for the expensive living costs in Jakarta during the surgery preparation. (2) Long waiting list; valve surgery has only been performed in a few hospitals in Indonesia. Almost all complex cases (including RHD) nationwide were referred to our center. Therefore, there was an imbalance between the number of patients and the available surgical schedules, causing a long waiting period (up to six months). (3) COVID era. People were reluctant to travel during the pandemic, especially to the hospital. (4) Social reason. Family concerns and fear of having cardiac surgery were the common reasons for them to delay surgery until the symptoms became more severe.

The durability of rheumatic MVr is a major concern, as several studies still report controversy [12,19,26,36]. It is known that repairing rheumatic MV is technically more complicated and challenging [13]. The problems occur either in young or older adult patients. In young patients, the ongoing rheumatic process may cause a higher risk of initial repair failure, which is also an independent predictor of reoperation and valve failure [36,37]. In old patients with RHD, extensive fibrosis, scarring, and calcification lead to retraction and stenosis on the leaflets, resulting in more challenges in valve repair and may compromise durability [38]. Chen et al. reported that a history of percutaneous transvenous mitral commissurotomy (PTMC) and not the type of MV lesion is a risk factor for reoperation [12]. We found that a history of PTMC was a risk factor for late mortality. Brescia et al. suggested improved outcomes correlated with a good evaluation of anterior leaflet mobility or calcification to decide on mitral valve repair or replacement [39]. Mitral reoperation in the RHD group was so few in our study. Since we could not monitor the valve condition using echocardiography during follow-up, the low MV reoperation cases did not necessarily suggest good repair durability. Further study with a specific design is needed to elaborate on that issue.

A study from a developed country reported that in the recent decade, the number of MVr is markedly reduced compared to the previous decade [34]. In the present time, the application of a valve in valve procedure may alter the threshold of mitral valve replacement for the decision-making of a surgical approach for MV surgery [39]. In contrast, most developing countries with low or low-middle income face similar issues, including limited access to health centers, insufficient experienced cardiac centers, especially for performing rheumatic valve surgery, and inadequate financial assistance from the government. Considering these circumstances, although in our study, MVr was not significantly superior to MVR, a trend toward better cumulative late survival may suggest it is a desirable surgical approach. As our center performs most valve surgery countrywide, the outcome can suggest a general overview of the state of rheumatic valve surgery in the country. This study did not aim to evaluate the correlation between the specific pathological features of RHD and the outcome. However, heavy calcification, fibrotic leaflets, small annulus, and thickening of subvalvular apparatus, which occur in advanced disease stages, predict failed repair. These pathologies in our study showed the advanced stage of the disease. Hence, we need to increase the awareness of the community about the disease, how to prevent the disease progression, and the importance of having early valve surgery to prevent more extensive surgical procedures and have a better outcome. Finally, whether the repair is a better surgical approach for rheumatic MV disease depends on multiple factors. More importantly, increasing understanding of the pathology, improving surgical technique, a high number of cases, and surgeons’ experience will eventually improve the clinical outcome of this disease [13].

In light of all the findings and discussion above, it is proven that RHD remains a significant health problem, especially in developing countries. It causes disability, poor quality of life, early mortality, and a national economic burden. The definitive management of RHD is surgery, which costs a lot. Since RHD is a preventable disease, secondary prophylaxis is important and most beneficial to the latent RHD group [40]. Hence, screening for the latent RHD group is essential to ease the burden of this disease. Developing an effective and efficient RHD screening program will enable us to increase coverage and reach a more screened population [41].

### Study limitations

The primary limitation of this study was the observational and retrospective nature of the study design. Some of our data was not normal in distribution. In real-life situations, not normally distributed data is not uncommon. To minimize the effect of non-normally distributed data, we performed suitable statistical tests to manage the data. The morphology of each valve lesion and the respective surgical techniques used were not included in the registry data. However, we reported the techniques for repairing the rheumatic mitral and non-rheumatic mitral lesion and the respective valve morphology, which encouraged the surgeon to repair the valve. This information could give a general condition of the morphology and surgical technique in our center, which may relate to the study outcome.

## Conclusion

The 30-day mortality rate in rheumatic valve surgery was higher than in non-rheumatic valve surgery. In rheumatic mitral valve disease, MV repair did not show a significant survival advantage over MVR, although a trend towards more favourable survival was detected. In non-rheumatic mitral valve disease, MV repair showed better survival than MV replacement.

## Additional Files

The additional files for this article can be found as follows:

10.5334/gh.1285.s1 Central Illustration. Comparison between surgical approach in both etiologic groups for early, late mortalities, and cumulative survival.

10.5334/gh.1285.s2 Supplementary File. Table Supplement 1 to 3.
