# Supplementary material for: Clinical Outcome of Rheumatic Mitral Valve Repair and Replacement Surgery in Indonesia; A Comparison with Non-Rheumatic Aetiology
Source: Glob Heart. 2024 Jan 11;19(1):4. doi: 10.5334/gh.1285 (PMC10786046; doi:10.5334/gh.1285)
Supplement: Central Illustration. — Comparison between surgical approach in both etiologic groups for early, late mortalities, and cumulative survival. [file gh-19-1-1285-s1.pdf]

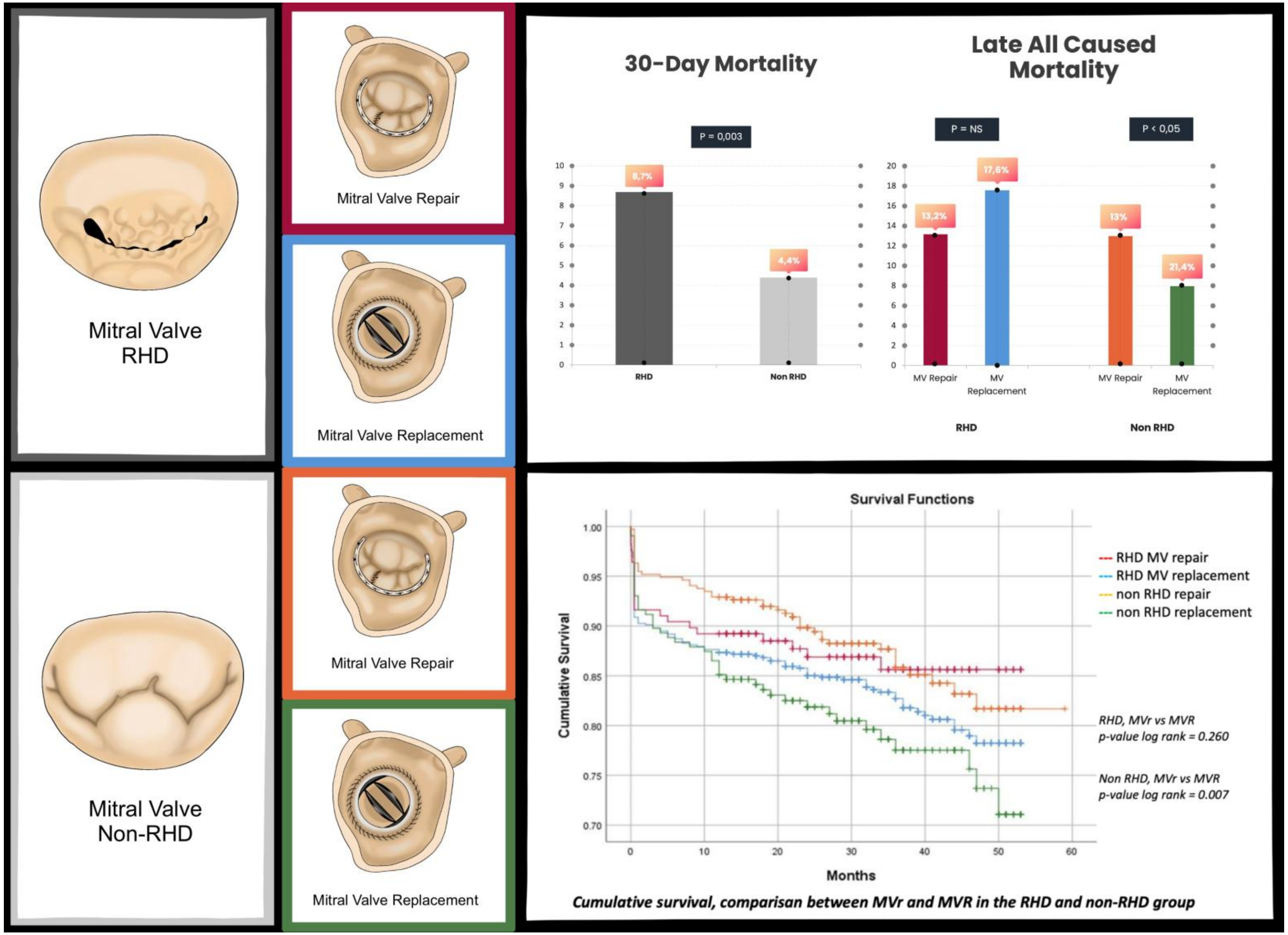

Central Illustration. Comparison between surgical approach in both etiologic groups for early, late mortalities, and cumulative survival.
