# Supplementary material for: Clinical Outcome of Rheumatic Mitral Valve Repair and Replacement Surgery in Indonesia; A Comparison with Non-Rheumatic Aetiology
Source: Glob Heart. 2024 Jan 11;19(1):4. doi: 10.5334/gh.1285 (PMC10786046; doi:10.5334/gh.1285)
Supplement: Supplementary File. — Table Supplement 1 to 3. [file gh-19-1-1285-s2.pdf]

Table Supplement 1. Basic characteristics of MV surgery patients based on etiology and surgical approach

Variables	Rheumatic Heart Disease			Non-Rheumatic Heart Disease			P value	p value	p value
	Total N = 814	Mitral Repair N= 167	Mitral Replace N= 647	Total N = 568	Mitral repair N = 353	Mitral Replace N = 215	RHD vs non RHD (total)	RHD Repair vs replace	Non-RHD Repair vs replace
Age	43 (18- 70)	41 (18-69)	43 (18-70)	54 (18-78)	54 (18 -78)	53 (18-73)	**<0.0001**	0.147	0.696
Female	515 (63.3%)	112 (67.1%)	403 (62.3%)	182 (32.0%)	121 (34.3%)	61 (28.4%)	**<0.0001**	0.293	0.171
BMI	21.9 (13.6-43.4)	22.1 (13.6-39.7)	21.8 (14.0-43.4)	23.1 (12.9-39.1)	23.5 (12.9-39.1)	22.2 (14.5-39.1)	**<0.0001**	0.444	**0.008**
**Preoperative Comorbidities**									
Atrial Fibrillation	587 (72.1%)	106 (63.5%)	481 (74.3%)	180 (31.7%)	106 (30%)	74 (34.4%)	**<0.0001**	**0.007**	0.318
DM with Insulin. n (%)	6 (0.7%)	1 (0.6%)	5 (0.8%)	6 (1.1%)	4 (1.1%)	2 (0.9%)	0.565	1.00	1.00
COPD. n (%)	5 (0.6%)	0 (0%)	5 (0.8%)	2 (0.4%)	0 (0%)	2 (0.9%)	0.707	0.589	0.143
Hypertension	81 (10%)	14 (8.4%)	67 (10.4%)	179 (31.5%)	117 (33.1%)	62 (28.8%)	**<0.0001**	0.539	0.328
CKD (CCL< 50 ml/m2)	119 (14.6%)	26 (15.6%)	93 (14.4%)	146 (25.7%)	80 (22.7%)	66 (30.7%)	**<0.0001**	0.790	**0.043**
NYHA fc I –II	412 (50.6%)	91 (54.5%)	321 (49.6%)	314 (55.3%)	202 (57.2%)	112 (52.1%)	0.098	0.300	0.269
NYHA fc III-IV	402 (49.4%)	76 (45.5%)	326 (50.4%)	254 (44.7%)	151 (42.8%)	103 (47.9%)	0.098	0.300	0.269
Infective Endocarditis	22 (2.7%)	5 (3%)	17 (2.6%)	44 (7.7%)	10 (2.8%)	34 (15.8%)	**<0.0001**	0.790	**<0.0001**
**Concomitant Cardiac Surgery/Intervention**									
CABG	27 (3.3%)	6 (3.6%)	21 (3.2%)	93 (16.4%)	48 (13.6%)	45 (20.9%)	**<0.0001**	1.00	**0.03**
Aortic Valve Surgery	238 (29.2%)	46 (27.5%)	192 (29.7%)	40 (7%)	18 (5.1%)	22 (10.2%)	**<0.0001**	0.657	**0.032**
Tricuspid Valve Surgery	472 (58%)	82 (49.1%)	390 (60.3%)	141 (24.8%)	60 (17%)	81 (37.7%)	**<0.0001**	**0.012**	**<0.0001**
$\geq$3 surgical procedures	134 (16.5%)	25 (15%)	109 (16.8%)	16 (2.8%)	4 (1.1%)	12 (5.6%)	**<0.0001**	0.641	**0.004**
History of PTMC	13 (1.6%)	3 (1.8%)	10 (1.5%)	0 (0%)	0 (0%)	0 (0%)	**<0.006**	0.736	**-**
CPB time	106 (41-390)	109 (44-251)	105.5 (41-390)	100 (10-515)	95 (10-261)	110 (45-515)	**0.002**	0.583	**<0.0001**
Aox time	80 (5-319)	78 (5-225)	80.5 (27-319)	76 (12-467)	72.50 (12-235)	82 (25-467)	**0.021**	0.976	**<0.0001**
**Cardiac condition (echo parameters)**									
LVEF (%)	60 (20-82)	60 (22-80)	60 (20-82)	66 (25-89)	67 (27-89)	65 (25-82)	**<0.0001**	0.591	**0.002**
TAPSE (mm)	18 (6-37)	19 (8-36)	18 (6.0-37.0)	23 (5.3-40.0)	24.0 (6.2-39.4)	22 (5.3-40.0)	**<0.0001**	**0.002**	0.129
TVG	41 (0-156)	37 (0-100)	42 (0-156)	31 (0-111)	30 (0-108)	33 (0-111)	**<0.0001**	0.073	**0.015**
Mitral stenosis	342 (42%)	69 (41.3%)	273 (42.2%)	0 (0%)	0 (0%)	0 (0%)	**<0.0001**	0.907	-
Mitral regurgitation	166 (20.4%)	52 (31.1%)	114 (17.6%)	568 (100%)	353 (100%)	215 (100%)	**<0.0001**	**<0.0001**	-
Mixed mitral valve disease	306 (37.6%)	46 (27.5%)	260 (40.2%)	0 (0%)	0 (0%)	0 (0%)	**<0.0001**	**0.004**	-
**Risk Assessment for Mortality**									
Euroscore II	2.0 (0.5-19.5)	1.9 (0.5-19.5)	2.1 (0.5-16.2)	1.6 (0.5-21.3)	1.4 (0.5-14.0)	2.2 (0.5-21.3)	**<0.0001**	**0.049**	**<0.0001**

DM: diabetes mellitus, COPD: Chronic Obstructive Pulmonary Disease, CKD: chronic kidney disease, NYHAfc: New York Heart Association functional class, CABG: coronary artery bypass graft, PTMC: percutaneous Transcatheter Mitral Valvotomy, CPB: cardiopulmonary bypass, AoX: aortic cross clem, LVEF: left ventricle ejection fraction, TAPSE: tricuspid annular plane systolic excursion, TVG: tricuspid valve gradient.

Table Supplement 2. Outcome following mitral valve surgery based on etiology and surgical approach

Variables	Rheumatic Heart Disease			Non-Rheumatic Heart Disease			P value	p value	p value
	Total N = 814	Mitral Repair N= 167	Mitral Replace N= 647	Total N = 568	Mitral repair N = 353	Mitral Replace N = 215	RHD vs non RHD (total)	RHD Repair vs replace	Non-RHD Repair vs replace
30 days mortality	71 (8.7%)	14 (8.4%)	57 (8.8%)	25 (4.4%)	12 (3.4%)	13 (6.0%)	**0.003**	0.984	0.200
30 days redo surgery	15 (1.8%)	4 (2.4%)	11 (1.7%)	9 (1.6%)	6 (1.7%)	3 (1.4%)	0.879	0.524	1.00
**Late outcome**									
Late all caused mortality	136 (16.7%)	22 (13.2%)	114 (17.6%)	92 (16.2%)	46 (13%)	46 (21.4%)	0.859	0.209	**0.012**
Late redo MV surgery	3 (0.4%)	0 (0%)	3 (0.5%)	5 (0.9%)	4 (1.1%)	1 (0.5%)	0.285	1.00	0.655

MV: mitral valve, RHD: rheumatic heart disease

Table Supplement 3. 30 days reoperation associated with late all caused mortality in patient MV Surgery

30 days redo surgery	Late all caused mortality		P value	HR (95%CI)
	Alive (N=1154)	Death (N=228)		
**Etiology**				
RHD	5 (33.3%)	10 (66.7%)	**<0.0001***	5.02 (2.63 – 9.58)
Non RHD	4 (44.4%)	5 (55.6%)	**<0.0001***	5.89 (2.38 – 14.59)

Data was analysed by Cox Regression. *Statistically significant (p value <0.05)
